# A Novel Major Pilin Subunit Protein FimM Is Involved in Adhesion of *Bifidobacterium longum* BBMN68 to Intestinal Epithelial Cells

**DOI:** 10.3389/fmicb.2020.590435

**Published:** 2020-11-23

**Authors:** Yao Xiong, Zhengyuan Zhai, Yuanqiu Lei, Bingbing Xiao, Yanling Hao

**Affiliations:** ^1^ Beijing Advanced Innovation Center for Food Nutrition and Human Health, College of Food Science and Nutritional Engineering, China Agricultural University, Beijing, China; ^2^ Key Laboratory of Functional Dairy, Co-constructed by Ministry of Education and Beijing Municipality, College of Food Science and Nutritional Engineering, China Agricultural University, Beijing, China; ^3^ Department of Obstetrics and Gynecology, Peking University First Hospital, Beijing, China

**Keywords:** adhesion, Bifidobacterium longum BBMN68, FimM, major pilin subunit protein, surface adhesin, adhesion receptors

## Abstract

Adhesion to the gastrointestinal tract is considered to be important for bifidobacteria to colonize the human gut and exert their probiotic effects. Some cell surface proteins of bifidobacteria, known as adhesins, play critical roles in the binding to host cells or the extracellular matrix (ECM). To elucidate the mechanisms associated with the adhesion of *Bifidobacterium longum* BBMN68, a centenarian originated potential probiotic, PSORTdb was employed to identify putative extracellular localized proteins in the *B. longum* BBMN68. Of the 560 predicted extracellular proteins, 21 were further identified as putative adhesion proteins using the conserved domain database of NCBI, and four were successfully overexpressed in the heterologous host, *Lactococcus lactis* NZ9000. Notably, a recombinant strain expressing FimM showed a significantly increased adhesive affinity for both HT-29 and mucus-secreting LS174T goblet cells (2.2- and 5.4-fold higher than that of the control strain, respectively). Amino acid sequence alignment showed that FimM is a major pilin subunit protein containing a Cna-B type domain and a C-terminal LPKTG sequence. However, *in silico* analysis of the *fimM*-coding cluster revealed that *BBMN68_RS10200*, encoding a pilus-specific class C sortase, was a pseudogene, indicating that FimM may function as a surface adhesin that cannot polymerize into a pili-like structure. Immunogold electron microscopy results further confirmed that FimM localized to the surface of *L. lactis* NZfimM and *B. longum* BBMN68 but did not assemble into pilus filaments. Moreover, the adhesive affinity of *L. lactis* NZfimM to fibronectin, fibrinogen, and mucin were 3.8-, 2.1-, and 3.1-fold higher than that of the control. The affinity of FimM for its attachment receptors was further verified through an inhibition assay using anti-FimM antibodies. In addition, homologs of FimM were found in *Bifidobacterium bifidum* 85B, *Bifidobacterium gallinarum* CACC 514, and 23 other *B. longum* strains by sequence similarity analysis using BLASTP. Our results suggested that FimM is a novel surface adhesin that is mainly present in *B. longum* strains.

## Introduction

Bifidobacteria are common inhabitants of the human gastrointestinal tract (GIT), constituting approximately 60–90% of the total gut microbiome in early life (Odamaki et al., 2016). Some bifidobacteria can confer health-promoting benefits on their human hosts, such as the competitive exclusion of pathogens, alleviation of inflammatory bowel disease symptoms, modulation of immune responses, and reduction of serum cholesterol levels (Gareau et al., 2010; Buffie and Pamer, 2013). Probiotics are “live microorganisms which, when administered in adequate amounts, confer a health benefit on the host” (Morelli, 2013). Some bifidobacteria fit into this category and are used as active ingredients in functional dairy-based products (Foligne et al., 2013). The adhesion of a probiotic bacterium to the host can increase its transit time in the gut, thereby enhancing its beneficial properties, such as the promotion of immunomodulatory effects and inhibition of pathogen adherence through competition for host cell binding sites (Tuo et al., 2018; Monteagudo-Mera et al., 2019). Consequently, the ability to adhere to human intestinal mucus and/or human intestinal epithelial cells is a commonly employed criterion for the selection of potential probiotics (Klaenhammer and Kullen, 1999; Tuomola et al., 2001).

Bifidobacteria generally employ surface adhesion proteins, including pili, moonlighting proteins, and other surface-anchored proteins, to adhere to the host GIT (Klijn et al., 2005; Westermann et al., 2016). For instance, in *Bifidobacterium bifidum* PRL2010, two sortase-dependent pili bind to Caco-2 cells and extracellular matrix (ECM) proteins such as fibronectin, plasminogen, and laminin (Turroni et al., 2013); the moonlighting proteins EF-Tu and enolase serve as surface adhesins for the binding of *Bifidobacterium longum* NCC2705 to human plasminogen and Caco-2 cells (Wei et al., 2014); BL0155, a large extracellular transmembrane protein isolated from *B. longum* VMKB44, is important for its binding to HT-29 epithelial cells *in vitro* (Shkoporov et al., 2008); *B. bifidum* ATCC 15696 employs a sialidase to mediate its adhesion to mucus (Nishiyama et al., 2017); in *B. longum* JCM1217, endo-*α*-*N*-acetylgalactosaminidase was reported to bind mucin (Suzuki et al., 2009); transaldolase was reported to act as a surface mucin-binding protein in several *B. bifidum* strains (Gonzalez-Rodriguez et al., 2012); and, in *Bifidobacterium animalis*, enolase, the chaperone protein DnaK, and the heat-shock protein GroEL were shown to bind plasminogen or Caco-2 cells (Candela et al., 2009, 2010; Sun et al., 2016). Combined, these observations indicate that bifidobacterial species differ from each other in their adhesion strategies, and there is marked variation in adhesion mechanisms even within individual species.

*Bifidobacterium longum* BBMN68 was isolated from a healthy centenarian in Bama County of Guangxi province, China, which is known for having a high life expectancy. BBMN68 has been reported to exert several potential probiotic functions, such as enhancing both innate and acquired immunity, alleviating allergic responses, and improving intestinal function (Yang et al., 2009, 2015). Because adhesion to the host is important for bifidobacteria to exert their health-promoting effects, in this study, we investigated the mechanisms that mediate the adhesion of *B. longum* BBMN68 to epithelial cells. For this, 9 of 21 predicted BBMN68 surface adhesion proteins were expressed in a heterologous host, *Lactococcus lactis* NZ9000. A novel pilin subunit protein – BBMN68_RS02235, designated FimM – was identified as a putative surface adhesion protein that mediates the adhesion of *B. longum* BBMN68 to mucin, fibronectin, and fibrinogen.

## Materials and Methods

### Bacterial Strains and Growth Conditions

The bacterial strains and plasmids used in this study are listed in Supplementary Table S1. *Bifidobacterium longum* BBMN68 cells (GenBank accession no. NC_014656.1) were grown anaerobically at 37°C in de Man-Rogosa-Sharpe (MRS) broth supplemented with 1% (*v/v*) L-cysteine (MRSc). *Lactococcus lactis* NZ9000 was grown at 30°C in M17 medium (Oxoid, Unipath, Basingstoke, United Kingdom) containing 0.5% (*w/v*) glucose (GM17). *Escherichia coli* strains were grown aerobically at 37°C in Luria-Bertani (LB) medium with shaking (220 rpm). When required, media were supplemented with the relevant antibiotics at the following concentrations: 100 μg ml^−1^ ampicillin and 10 μg ml^−1^ chloramphenicol for *E. coli*, and 5 μg ml^−1^ chloramphenicol for *L. lactis*.

### The Heterologous Expression of Putative Surface Adhesion Proteins in *L. lactis* NZ9000

Genomic DNA was extracted from *B. longum* BBMN68 using a TIANamp Bacteria DNA Kit according to the manufacturer’s instructions (TianGen, Beijing, China). Nine genes encoding predicted surface adhesion proteins were amplified from genomic DNA using the primer pairs listed in Supplementary Table S2. The PCR products digested with *Kpn*I/*Hin*dIII or *Kpn*I/*Xba*I (NEB, Beijing, China) were inserted into the corresponding sites in pNZ81481. The ligated plasmids were then transformed into *L. lactis* NZ9000 by electroporation using Bio-Rad Gene Pulser Xcell (Bio-Rad, Richmond, CA, United States) as previously described (DeRuyter et al., 1996). The resulting recombinant plasmids were isolated using the E.Z.N.A. Plasmid Mini Kit I (Omega Bio-tek Inc., Doraville, GA, United States). The recombinant strains were confirmed by plasmid sequencing and further analyzed with the DNAMAN software package. Meanwhile, *L. lactis* NZCK harboring the empty pNZ81481 vector was used as the control strain. Overnight cultures of the recombinant strains were inoculated (1% inocula) into 10 ml of fresh GM17 medium containing 5 μg ml^−1^ chloramphenicol. To induce gene expression, when the cell density had reached an OD_600_ nm of 0.2~0.3, the cultures were supplemented with 10 ng ml^−1^ nisin (Sigma-Aldrich, Milwaukee, WI, United States) and incubated for an additional 2 h at 30°C. The cell pellets were then collected after centrifugation at 6,000 × *g* for 5 min at 4°C for subsequent SDS–polyacrylamide gel electrophoresis (SDS–PAGE) analysis and adhesion assays.

### Bacterial Adhesion to HT-29 and LS174T Cells

The human colon adenocarcinoma cell line HT-29 and the goblet cell-derived cell line LS174T were obtained from the China Infrastructure of Cell Line Resource. The cells were cultured at 37°C in a humidified atmosphere with 5% CO_2_. The HT-29 cells were grown in Dulbecco’s high-glucose modified Eagle’s medium (DMEM, Thermo Fisher Scientific, Rockford, IL, United States) supplemented with 10% (*v/v*) fetal bovine serum (FBS, Invitrogen, New York, NY, United States) and 100 U ml^−1^ penicillin/streptomycin (Gibco, Waltham, MD, United States); the mucus-producing LS174T cells (Hews et al., 2017) were grown in RPMI 1640 medium (Gibco) supplemented with 2 mM L-glutamine (Gibco), 10% FBS, and 100 U ml^−1^ penicillin/streptomycin. The cells were subcultured every 2–3 days. For adhesion analysis, cells were seeded at a density of 1 × 10^5^ cells per well into 24-well plates and grown to ~90% confluence. Epithelial cell monolayers were carefully washed twice with phosphate-buffered saline (PBS, pH 7.4) before the addition of bacterial cells. Recombinant *L. lactis* strains were washed twice with PBS and resuspended in DMEM or RPMI 1640 medium without antibiotics at a concentration of ~1 × 10^7^ colony-forming units (CFUs) ml^−1^. Aliquots (1 ml) of *L. lactis* suspension were added to the wells. The plates were incubated for 1 h at 37°C in 5% CO_2_, following which the wells were gently washed five times with PBS to remove unattached bacteria. The epithelial cells with adherent bacteria were detached using 0.25% trypsin (Sigma-Aldrich, St. Louis, MO, United States) treatment. Bacterial counts were determined by plating 10-fold serial dilutions on GM17 plates. Adhesion ratios were calculated as a percentage using the following formula: 100 × the number of adherent bacteria/the number of bacteria inoculated. The results were representative of three independent experiments, each performed in triplicate.

### Bacterial Adhesion to Mucin and ECM Proteins

The extracellular matrix proteins (fibronectin, laminin, collagen I, collagen IV, fibrinogen, and plasminogen; Sigma-Aldrich, St. Louis, MO, United States; product codes: 10838039001, L4544, C7624, C5533, F3879, and P7999, respectively), were all of human origin. Mucin (Sigma-Aldrich, St. Louis, MO, United States; product code: M2378) was isolated from the porcine stomach. The ECM proteins and mucin were immobilized in wells of a Nunc Maxisorp 96-well microplate (Maxisorp; Nunc, Roskilde, Denmark) by overnight incubation at 4°C at a concentration of 2.5 pmol well^−1^. The wells were then washed twice with PBS and incubated with blocking buffer [2% (*w/v*) BSA in PBS] for 2 h at 37°C. The wells were subsequently washed three times with PBS before adding the bacteria. The bacteria were washed twice with PBS and resuspended in PBS to a final OD_600_ equivalent to ~1 × 10^8^ CFUs ml^−1^. Aliquots (200 μl) of *L. lactis* or *B. longum* suspension were added to coated 96-well plates and incubated at 30°C (*L. lactis*) or 37°C (*B. longum*) for 1 h. The unattached bacteria were removed by washing the wells three times with PBS. Bacteria bound to ECM proteins were detached by treatment with PBS containing 0.01% (*v/v*) Triton X-100 followed by incubation at 30 or 37°C for 30 min with shaking (200 rpm). The bacterial counts were determined on GM17 (*L. lactis*) or MRSc (*B. longum*) plates. The adhesion ratios, expressed as percentages, were calculated by comparing the bacterial counts after adhesion to the number of cells in the bacterial suspension added originally to the plate wells. All the results were representative of three independent experiments, each performed in triplicate. For the inhibition assays, *L. lactis* and *B. longum* BBMN68 were preincubated with anti-FimM antibody (diluted 1:10,000 in PBS) for 1 h, after which the protocol was continued as outlined above.

### Generation of the Polyclonal Anti-FimM Antiserum and Antibodies

The *fimM* fragment, without the regions encoding the signal sequence and the LPxTG motif, was amplified from *B. longum* BBMN68 using the primer pair *fimM-GST*-F and *fimM-GST*-R (Supplementary Table S2). The predicted product was cloned into the pGEX-4T-1 vector (GE Healthcare, Madison, WI, United Kingdom) for expression as an N-terminal GST-fusion protein in *E. coli* BL21 (DE3). When the OD_600_ of the recombinant cells had reached 0.4, isopropyl-*β*-D-thiogalactopyranoside (IPTG) was added to induce protein expression. Soluble proteins were then purified by glutathione-sepharose 4B affinity chromatography (Solarbio, Beijing, China) after a 12 h incubation at 16°C. Subsequently, the GST tag of the purified protein was removed by thrombin cleavage (Solarbio). The purified proteins were detected by SDS-PAGE and the concentration was measured using a NanoDrop 2000 spectrophotometer (Thermo Fisher Scientific, Wilmington, DE, United States). Polyclonal antibodies against FimM were raised in rabbits by BIOSS CO., LTD (Beijing, China) as previously described (Johnston et al., 1991).

### Immunogold Electron Microscopy

*Lactococcus lactis* NZCK, *L. lactis* NZfimM, and the overnight-cultured *B. longum* BBMN68 were washed three times with PBS and then diluted (OD_600_ of 2.0) in the same buffer. Formvar/carbon-coated copper grids were floated for 10 min on droplets of the diluted cells in PBS, washed several times with the same buffer, and then treated with a blocking solution containing 1% bovine serum albumin (BSA) at 37°C for 30 min. The grids were then floated for 1 h on droplets of blocking solution containing anti-FimM serum (diluted 1:100), washed five times with 0.1% BSA in PBS to remove unbound antibodies, and incubated at 37°C for 1 h with 10-nm diameter gold particle-conjugated protein A diluted 1:55 in blocking solution. After several washes in PBS, the grids were negatively stained with a mixture of 1.8% methylcellulose-0.4% uranyl acetate. The samples were examined under a JEM-1400 transmission electron microscope (JEOL, Ltd., Tokyo, Japan).

### Statistical Analysis

Data were analyzed using GraphPad Prism 6 for Windows (GraphPad Software, Inc., La Jolla, CA, United States). When two groups were compared, an unpaired Student’s *t*-test was used to calculate *p* values.

## Results

### The Prediction of the Surface Adhesive Proteins of *B. longum* BBMN68

Using PSORTdb, a subcellular localization database for bacteria and archaea, 560 extracellular proteins were predicted to be present in the *B. longum* BBMN68 genome (Peabody et al., 2016). These included 507 proteins that localized to the cytoplasmic membrane and 25 cell wall-anchored proteins (Supplementary Table S3). For adhesion protein prediction, 21 proteins with possible adhesive functions were identified using NCBI’s conserved domain database (Marchler-Bauer et al., 2017; Table 1), including eight that contained a Gram-positive pilin subunit domain, two with a laminin G domain, seven that belonged to the glycosyl hydrolase family, two permeases belonging to the ABC-type transport system, one containing a cadherin-like beta-sandwich domain, and one S-layer protein.

**Table 1:** Putative surface adhesion proteins^a^ of *Bifidobacterium longum* BBMN68.

Locus tag	Gene name	Length of ORF^b^ (nt)	Protein definition	Domains predicted by CDD	SCL^c^
BBMN68_RS04640	*dppB3*	978	ABC transporter permease	ABC-type dipeptide/oligopeptide/nickel transport system, permease component	Membrane
BBMN68_RS05575	*potB*	951	Putative spermidine/putrescine transport system permease protein	ABC-type uncharacterized transport system, permease component	Membrane
BBMN68_RS05880	-	3,717	Probable extracellular protein possibly involved in xylan or arabinan degradation	Glycosyl hydrolase family 43; bacterial Ig-like domain (group 4)	Cell wall
BBMN68_RS05885	-	5,832	Hypothetical protein possibly involved in xylan degradation	F5/8 type C domain; bacterial Ig-like domain (group 4)	Cell wall
BBMN68_RS07375	-	3,741	Putative beta-xylosidase	Concanavalin A-like lectin/glucanases superfamily; bacterial Ig-like domain (group 4)	Cell wall/Extracellular
BBMN68_RS00335	*pgpB1*	1,530	Phosphatase PAP2 family protein	PAP2, bacterial acid phosphatase or class A non-specific acid phosphatase	Unknown
BBMN68_RS06090	*aprE*	5,901	Endo-alpha-N-acetylgalactosaminidase	Carboxypeptidase regulatory-like domain; endo-alpha-N-acetylgalactosaminidase; F5/8 type C domain	Extracellular
BBMN68_RS07145	*fimA*	1,578	Isopeptide-forming domain-containing fimbrial protein	Gram-positive pilin backbone subunit 2, Cna-B-like domain; LPXTG-motif cell wall anchor domain	Cell wall
BBMN68_RS07370	-	3,294	Hypothetical protein	Glycosyl hydrolase family 43; bacterial Ig-like domain (group 4)	Cell wall
BBMN68_RS07380	-	6,003	Hypothetical protein	Laminin G domain; bacterial Ig-like domain (groups 2 and 4)	Cell wall
BBMN68_RS07385	-	4,995	Hypothetical protein	Laminin G domain; glycosyl hydrolase family 43	Cell wall/Extracellular
BBMN68_RS09380	-	2,385	Hypothetical protein	Sortase domain found in class C sortases	Unknown
BBMN68_RS02235	*fimM*	1875	LPXTG cell wall anchor domain-containing protein	Gram-positive pilin backbone subunit 2, Cna-B-like domain; uncharacterized surface-anchored protein	Cell wall
BBMN68_RS06265	*tadE*	387	Pilus assembly protein TadE	Helicase/secretion neighborhood TadE-like protein	Unknown
BBMN68_RS06270	*tadF*	387	Pilus assembly protein	TadE-like protein	Membrane
BBMN68_RS06495	-	2,481	Hypothetical protein	Bacterial Ig-like domain (group 4); bacterial surface protein containing an Ig-like domain	Extracellular
BBMN68_RS09410	-	4,836	Hypothetical protein	Right-handed beta-helix region	Cell wall/Extracellular
BBMN68_RS07430	-	3,837	Hypothetical protein	Cadherin-like beta-sandwich domain; glycosyl hydrolase family 43; bacterial Ig-like domain (groups 3 and 4)	Cell wall/Extracellular
BBMN68_RS00860	*lspA*	549	Lipoprotein signal peptidase	Lipoprotein signal peptidase	Membrane
BBMN68_RS07365	-	3,198	Hypothetical protein	Glycosyl hydrolase family 43; bacterial Ig-like domain (groups 2 and 3)	Cell wall
BBMN68_RS04435	*slpA*	1,176	Hypothetical protein	Uncharacterized conserved protein; S-layer domain	Unknown

a Putative surface adhesion proteins, surface proteins containing domains have been reported to be involved in adhesion.

b ORF, open reading frame.

c SCL, subcellular location.

### The Expression of the Predicted Surface Adhesion Proteins in *L. lactis* NZ9000

**Figure 1:**
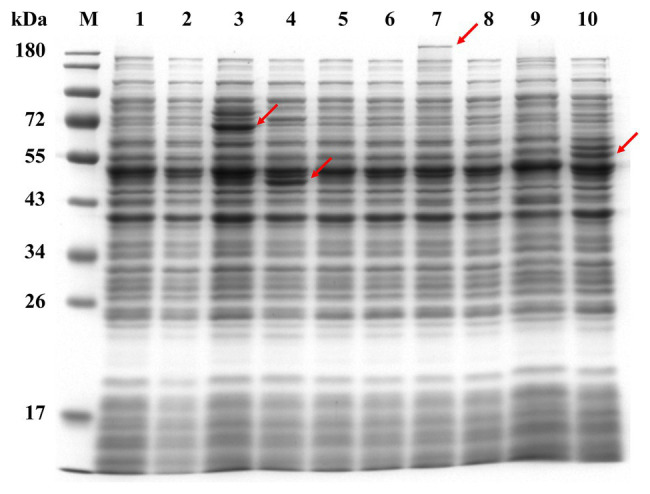
The heterologous expression of nine predicted surface adhesins detected by SDS-PAGE; soluble extracts were analyzed by denaturing SDS-PAGE (12%). Lane M, dual-color-prestained broad-molecular-size protein markers (10–180 kDa); lane 1, *Lactococcus lactis* NZCK; lane 2, *L. lactis* NZlspA; lane 3, *L. lactis* NZfimM; lane 4, *L. lactis* NZslpA; lane 5, *L. lactis* NZdppB3; lane 6, *L. lactis* NZpotB; lane 7, *L. lactis* NZaprE; lane 8, *L. lactis* NZtadE; lane 9, *L. lactis* NZtadF; and lane 10, *L. lactis* NZfimA. Red arrows indicate the proteins overexpressed in each sample.

Although 21 genes in the *B. longum* BBMN68 genome were predicted to encode surface adhesion proteins, only 9 (*lspA*, *fimM*, *slpA*, *dppB3*, *potB*, *aprE*, *tadE*, *tadF*, and *fimA*; Table 1) were successfully cloned into the pNZ81481 expression vector (Supplementary Table S1). The remaining 12 genes could not be ligated into the vector possibly because these genes were too large for PCR amplification or ligation. After sequencing, the correct plasmids (respectively designated as pNZlspA to pNZfimA) were then transformed into *L. lactis* NZ9000 to generate recombinant strains (designated as *L. lactis* NZlspA to *L. lactis* NZfimA; Supplementary Table S1). SDS-PAGE analysis of total protein identified the overproduction of 64-, 41-, 211-, and 56-kDa proteins (Figure 1), which corresponded to the expected sizes of FimM, SlpA, AprE, and FimA, respectively. These results demonstrated that these four proteins could be successfully expressed in *L. lactis* NZ9000.

### The Adhesion of the Recombination Strains to HT-29 and LS174T Cells

**Figure 2:**
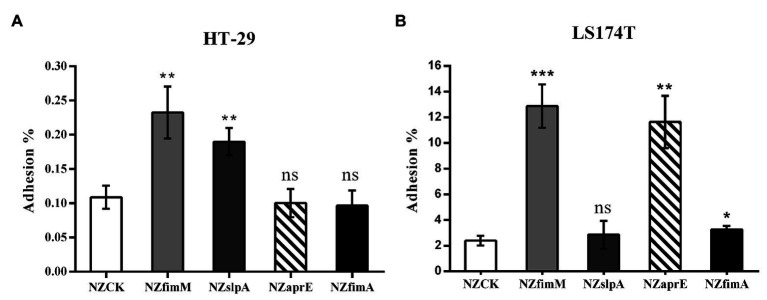
The adhesion of four recombinant strains and *L. lactis* NZCK to HT-29 **(A)** and LS174T **(B)** cells. The overexpression of FimM, SlpA, AprE, and FimA was induced by the administration of 10 ng ml^−1^ nisin before the adhesion assay. Adhesion percentages were calculated by dividing the number of colony-forming units (CFUs) of adherent bacteria by that of the initially added bacteria. Data represent the means ± standard deviation (SD) of three independent experiments. Significant differences between *L. lactis* NZCK (negative control) and each of the recombination strains were identified using an unpaired Student’s *t*-test. ns, not significant; ^*^p < 0.05; ^**^p < 0.01; and ^***^p < 0.001.

To investigate whether the expression of FimM, SlpA, AprE, and FimA could influence the adhesion of the host strain to intestinal epithelial cells, we performed adhesion assays between HT-29 and mucus-secreting LS174T cells (Walsham et al., 2016). The results showed that the adhesion ratios of strains *L. lactis* NZfimM and *L. lactis* NZslpA to HT-29 cells were 0.24 and 0.19%, respectively, which were 2.2- and 1.7-fold higher than that of the control *L. lactis* NZCK strain (Figure 2A). In addition, the adhesion ratios of strains *L. lactis* NZfimM, *L. lactis* NZaprE, and *L. lactis* NZfimA to LS174T cells were 12.87, 11.63, and 3.25%, values that were 5.4-, 4.9-, and 1.4-fold higher than that for *L. lactis* NZCK, respectively (Figure 2B). Notably, only the overexpression of FimM in *L. lactis* NZ9000 led to a significant increase in adhesion to both cell types. Consequently, we subsequently focused on investigating the adhesion mechanisms associated with FimM.

### The Cell Surface Localization of FimM in *L. lactis* NZfimM

**Figure 3:**
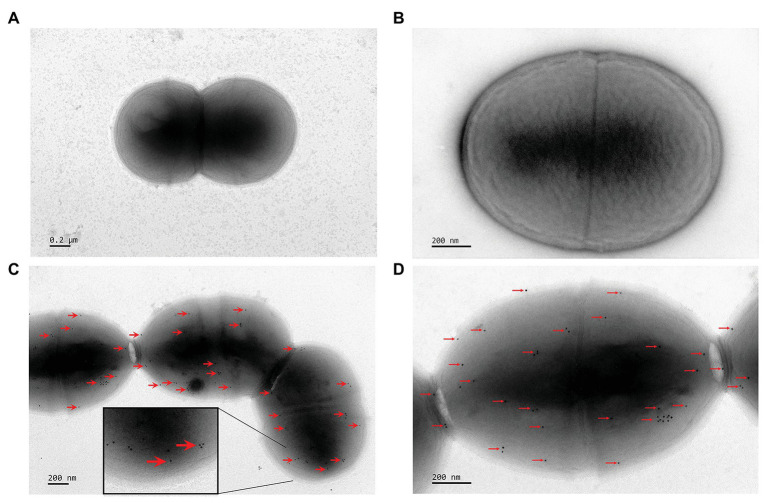
The visualization of cell surface-localized FimM in *L. lactis* NZCK and *L. lactis* NZfimM. Immunogold labeling with anti-FimM serum and electron microscopy analysis of the *L. lactis* NZCK **(A,B)** and *L. lactis* NZfimM **(C,D)** strains. Arrows indicate gold particle labeling of FimM proteins. Scale bars, 200 nm.

To detect the localization of FimM at the surface of *L. lactis* NZfimM, cells of this strain were visualized by immunogold transmission electron microscopy. First, FimM was expressed as a GST-fusion in *E. coli* and purified by glutathione-sepharose 4B affinity chromatography. The GST tag was subsequently removed by thrombin cleavage. The purified protein showed an expected molecular mass of 58 kDa (Supplementary Figure S1), and was used to raise anti-FimM antiserum in rabbits. The specificity of the anti-FimM polyclonal antiserum was tested by western blotting. The results showed that the antiserum was specific for FimM, and showed no recognition for the total protein of NZfimM (Supplementary Figure S2). Subsequently, *L. lactis* NZfimM and *L. lactis* NZCK (negative control) cells were visualized by immunogold transmission electron microscopy using anti-FimM antiserum in combination with 10-nm gold particle-labeled protein A. A high number of gold particles were observed on the cell surface of *L. lactis* NZfimM (Figures 3C,D), whereas none were found on the negative control (Figures 3A,B), indicating that FimM localized to the surface of *L. lactis* NZfimM cells.

### The Adhesion Receptors for *B. longum* BBMN68 FimM

**Figure 4:**
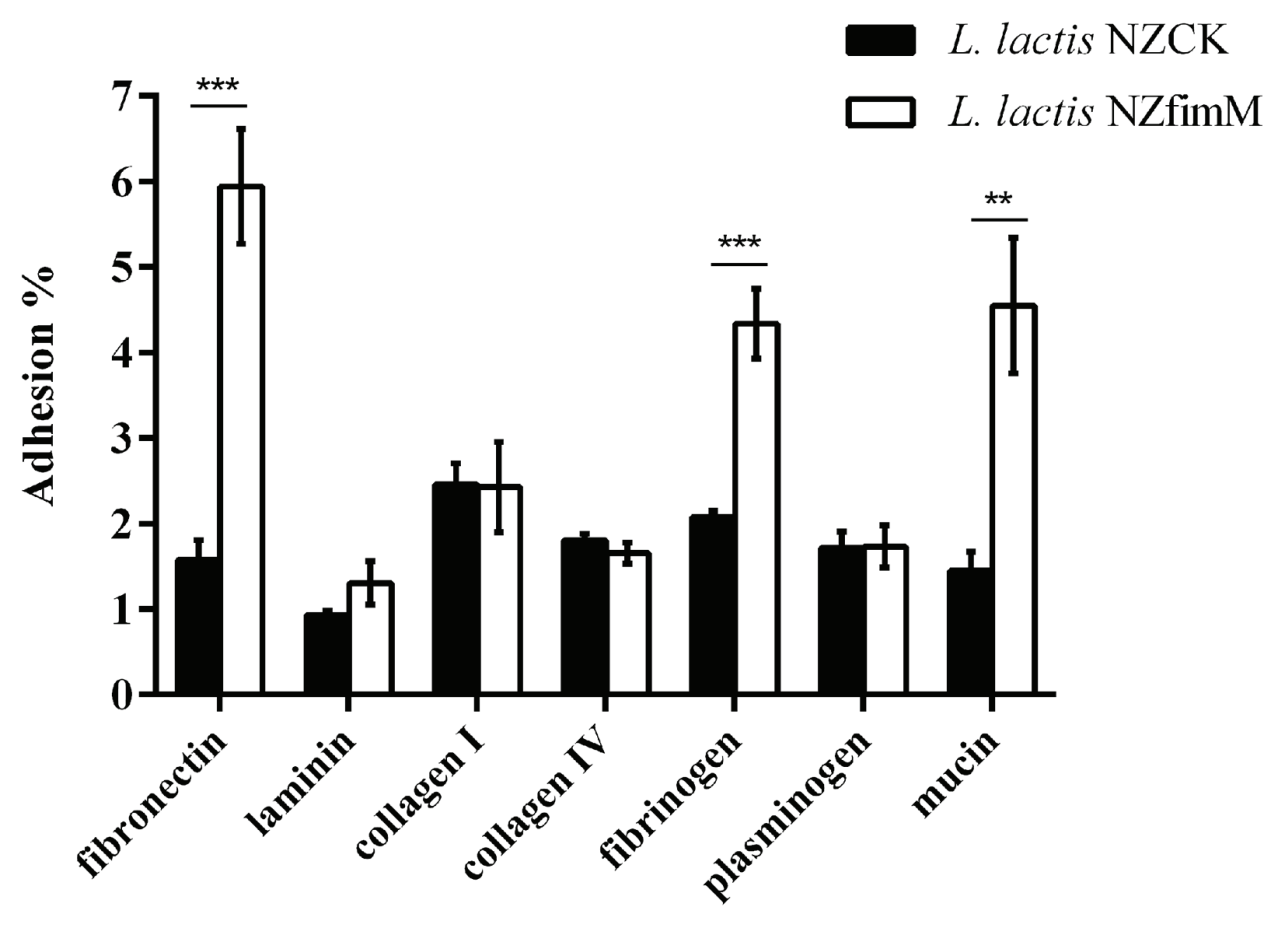
The adhesion of *L. lactis* NZCK and *L. lactis* NZfimM cells to mucin and various extracellular matrix (ECM) substrates following nisin induction. Adhesion percentages were calculated by dividing the number of CFUs of adherent bacteria by that of the initially added bacteria. Data represent the means ± SD of three independent experiments. Significant differences between *L. lactis* NZCK and *L. lactis* NZfimM were identified using an unpaired Student’s *t*-test. ^**^p < 0.01 and ^***^p < 0.001.

To identify the adhesion receptors involved in FimM recognition, we assessed the capacity of *L. lactis* NZfimM to adhere to mucin and the ECM substrates fibronectin, laminin, collagen type I, collagen type IV, fibrinogen, and plasminogen. The adhesion ratios of the control strain *L. lactis* NZCK for fibronectin, laminin, collagen type I, collagen type IV, fibrinogen, plasminogen, and mucin were 1.58, 0.93, 2.46, 1.81, 2.08, 1.72, and 1.45%, respectively. The corresponding adhesion ratios for *L. lactis* NZfimM were 5.94, 1.31, 2.43, 1.65, 4.34, 1.73, and 4.55%. These results showed that the adhesive affinity of *L. lactis* NZfimM for fibronectin, fibrinogen, and mucin was 3.8-, 2.1-, and 3.1-fold that of the control. No significant differences were observed between *L. lactis* NZfimM and the control strain for laminin, collagen I, collagen IV, and plasminogen binding (Figure 4). These results indicated that fibronectin, fibrinogen, and mucin are the adhesion receptors for the *B. longum* BBMN68 FimM protein.

### Inhibition Assays

**Figure 5:**
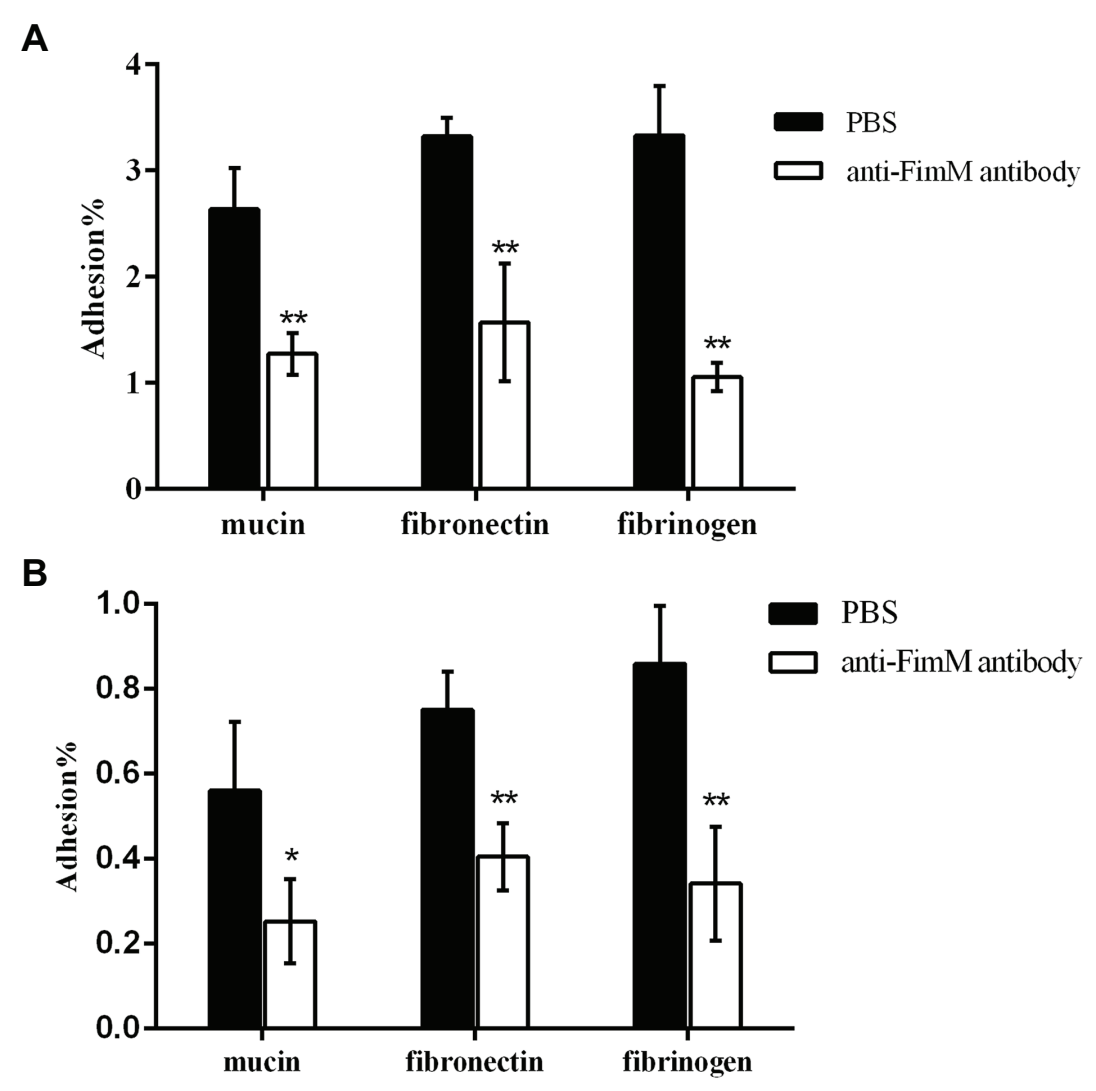
The inhibition of *L. lactis* NZfimM **(A)** and *B. longum* BBMN68 **(B)** adhesion to mucin, fibronectin, and fibrinogen with an anti-FimM antibody treatment. **(A)** *L. lactis* NZfimM cells were pretreated with phosphate-buffered saline (PBS; black columns) or an anti-FimM antibody (white columns) before being added to immobilized mucin, fibronectin, and fibrinogen for an adhesion assay. **(B)** Adhesion of *B. longum* BBMN68 to mucin, fibronectin, and fibrinogen in the presence (white columns) or absence (black columns) of the anti-FimM antibody. Data represent the means ± SD of three independent experiments. Significant differences between the PBS- and anti-FimM antibody-treated strains were identified using an unpaired Student’s *t*-test. ^*^p < 0.05 and ^**^p < 0.01.

To further confirm the specific binding of FimM to its recognition factors, we performed antibody-mediated inhibition experiments. *Lactococcus lactis* NZfimM cells were incubated with an anti-FimM antibody before being added to immobilized mucin, fibronectin, and fibrinogen. The results showed that the binding of NZfimM to mucin, fibronectin, and fibrinogen was reduced by 51.6, 52.6, and 68.3%, respectively (Figure 5A). Subsequently, *B. longum* BBMN68 cells were pretreated with an anti-FimM antibody. Notably, pretreated *B. longum* BBMN68 cells exhibited average reductions in their adhesion to mucin, fibronectin, and fibrinogen of 54.8, 46.1, and 87.8%, respectively (Figure 5B). These results provided further evidence that mucin, fibronectin, and fibrinogen function as adhesion receptors for FimM.

## Discussion

*In silico* analysis revealed that the *fimM*-coding cluster comprises *fimM*, encoding the major pilin subunit protein, and *BBMN68_RS10200*, which encodes a pilus-specific class C sortase (Supplementary Figure S4A). Amino acid sequence alignment showed that FimM contains an N-terminal signal peptide, a canonical C-terminal LPKTG sequence, and an E-box motif (YTFTEAKAPEGY; residues 522–533; Supplementary Figure S4B). However, FimM lacks the typical pilin motif that is involved in the covalent linkage between backbone subunits. Furthermore, sequence alignment of the BBMN68_RS10200 protein with the reference class C sortase, WP_007052877, showed that BBMN68_RS10200 lacks the canonical C-terminal region (179-242aa). Consequently, the Prokaryotic Genome Analysis Pipeline (PGAP) annotated *BBMN68_RS10200* as a pseudogene. We speculated that FimM is secreted under the guidance of the N-terminal signal peptide, and then the housekeeping sortase A cleaves the LPKTG motif and links the cleaved product to the cell wall (Swaminathan et al., 2007). However, FimM cannot polymerize into a multiprotein structure. This hypothesis was further confirmed by immunoelectron microscopy, where no pili-like structures were observed on the surface of *B. longum* BBMN68 (Supplementary Figure S3). The major pilin protein monomer BL0675 in *B. longum* subsp. *longum* 1-1 was reported as exhibiting a high adhesive affinity for mucin, but this ability was significantly reduced by treatment with anti-BL0675 antibodies (Suzuki et al., 2016). The recombinant major pilin protein monomer AafA also showed a marked binding affinity for fibronectin in enteroaggregative *E. coli* (Farfan et al., 2008). These results suggest that FimM, a major pilin subunit protein in *B. longum* BBMN68, may function as a surface adhesion monomer that cannot polymerize into pili-like structures.

Functional domain analysis revealed that FimM harbors a Cna-B type domain (residues 273–428; Supplementary Figure S4C), which is an immunoglobulin (IgG)-like domain initially identified in the collagen-binding protein (Cna) of *Staphylococcus aureus* (Deivanayagam et al., 2000). Cna-B domains have also been found in several pilins derived from Gram-positive bacteria, including FctB from *Streptococcus pyogenes*, RrgA and RrgC from *Streptococcus pneumoniae*, BcpA from *Bacillus cereus*, FimA and FimP from *Actinomyces naeslundii*, and SpaA from *Corynebacterium diphtheriae* (Krishnan, 2015). In *S. aureus*, the LDF motif of the Cna-B domain was shown to be an integrin-binding motif (Campbell and Humphries, 2011). In *Staphylococcus saprophyticus*, the CnaB-truncated mutant of the surface adhesin UafA displayed no ligand-binding activity, indicating that the Cna-B domain was necessary for host binding (Matsuoka et al., 2011). In *Actinomyces oris* T14V, the isolated Cna-B domain of the fimbrial shaft adhesin FimA can adhere to human oral epithelial (KB) cells as well as to glycoprotein asialofetuin (Mishra et al., 2011). In *B. bifidum* PRL2010, the major pilin subunit FimA, which contains a Can-B type domain, can bind to human ligands such as fibronectin, plasminogen, and laminin (Turroni et al., 2013). Taken together, these results indicate that the Cna-B domain may be the functional adhesion domain in FimM.

Pili and pilin proteins are typical adhesins that improve the colonization ability of orally administered probiotics (O’Connell Motherway et al., 2011; Aleksandrzak-Piekarczyk et al., 2016). However, growth conditions are important environmental factors influencing pilin expression levels in *Bifidobacterium* strains. The expression of the pilus gene clusters in *Bifidobacterium adolescentis* 22L is enhanced in response to starch, cellobiose, or maltodextrin *in vitro* (Duranti et al., 2014). Furthermore, the expression of the *tad* locus of *Bifidobacterium breve* UCC2003 and that of the *pil2* and *pil3* clusters of *B. bifidum* PRL2010 is enhenced in the murine GIT (O’Connell Motherway et al., 2011; Turroni et al., 2013). In our study, FimM was present in very low numbers on the cell surface of *B. bifidum* BBMN68, indicating that it was expressed at a low level (Supplementary Figure S3). We previously reported that the adhesion ratio of *B. longum* BBMN68 to HT-29 cells was 0.03% under *in vitro* growth conditions. However, the adhesive capacity of *B. longum* BBMN68 was increased 5-fold when these bacteria were grown with 0.075% (*w/v*) ox-bile (An et al., 2014), indicating that non-optimal *in vitro* growth conditions may result in a low level of FimM expression, thereby reducing the adhesive potential of *B. longum* BBMN68.

In this study, mucin, fibronectin, and fibrinogen were identified as the adhesion receptors for FimM. Mucins are the main structural components of the mucus layer that provides a physical barrier on the surface of the intestinal epithelium (Etzold and Juge, 2014). The ECM is a relatively stable structure that underlies the intestinal epithelium, and is mainly composed of fibronectin, laminin, collagen IV, plasminogen, and fibrinogen (Yadav et al., 2017). *Lactobacillus fermentum* can inhibit the adhesion of enteric pathogens, such as *E. coli* and *Proteus vulgaris*, by competing for mucin attachment sites (Chatterjee et al., 2018). During pathogen invasion, host mucosae are damaged and the ECM exposed, leading to infection. Under these conditions, the surface adhesins of probiotic bacteria can prevent pathogen adhesion to ECM components. For instance, the Cpb protein of *Lactobacillus plantarum* 91 was reported to play a key role in inhibiting *E. coli* 0157:H7/collagen interaction (Yadav et al., 2013). This suggests that, under normal circumstances, FimM may block pathogen access to the mucus layer by binding to mucins. Under pathogen invasion, FimM could competitively inhibit pathogen adhesion by binding to fibronectin and fibrinogen. Sequence homology analysis revealed that FimM is conservatively present in 26 *Bifidobacterium* strains (Supplementary Table S4). Comparative *in silico* analysis of the FimM derived from these strains showed more than 90% identity (Supplementary Figure S5). Notably, 24 of these 26 strains were strains of *B. longum* strains, indicating that FimM is a novel surface adhesin that is primarily present in strains of this bacterial species.

## Data Availability Statement

The original contributions presented in the study are included in the article/Supplementary Material, and further inquiries can be directed to the corresponding author.

## Ethics Statement

The animal study was reviewed and approved by Bioss Laboratory Animal Welfare and Animal Experiment Ethics Review Committee.

## Author Contributions

YH and YX designed the study and wrote the manuscript. YX and YL performed the experiments. YX analyzed and evaluated the data. YH and ZZ revised the manuscript. All authors read and approved the final version of the manuscript.

### Conflict of Interest

The authors declare that the research was conducted in the absence of any commercial or financial relationships that could be construed as a potential conflict of interest.
