# Supplementary material for: A Novel Major Pilin Subunit Protein FimM Is Involved in Adhesion of Bifidobacterium longum BBMN68 to Intestinal Epithelial Cells
Source: Front Microbiol. 2020 Nov 23;11:590435. doi: 10.3389/fmicb.2020.590435 (PMC7719627; doi:10.3389/fmicb.2020.590435)
Supplement: Supplementary file 1 [file Data_Sheet_1.pdf]

Supplementary Material

1.1 Supplementary Figures

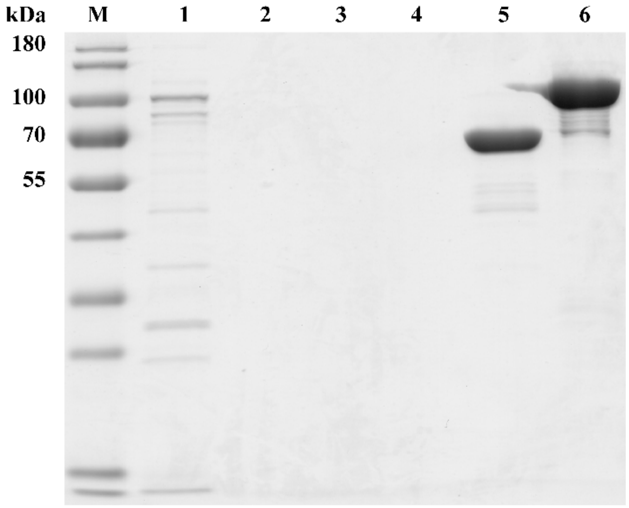

Supplementary Figure S1. SDS-PAGE analysis of purified recombinant FimM protein. Lane M, dual-color-prestained broad-molecular-size protein markers (10 to 180 kDa); lane 1, flow-through buffer; lane 2,3 and 4, wash buffer; 5: purified FimM protein; 6: purified FimM-GST fusion.

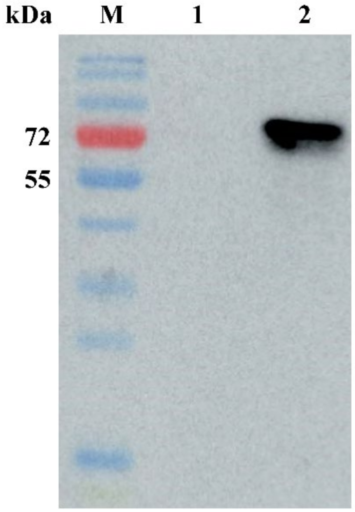

Supplementary Figure S2 Western blot analyses of total proteins from *L. lactis* NZCK (lane 1) and *L. lactis* NZfimM (lane 2). Membranes were immunoblotted with anti-FimM serum. Lane M, dual-color-prestained broad-molecular-size protein markers (10 to 180 kDa).

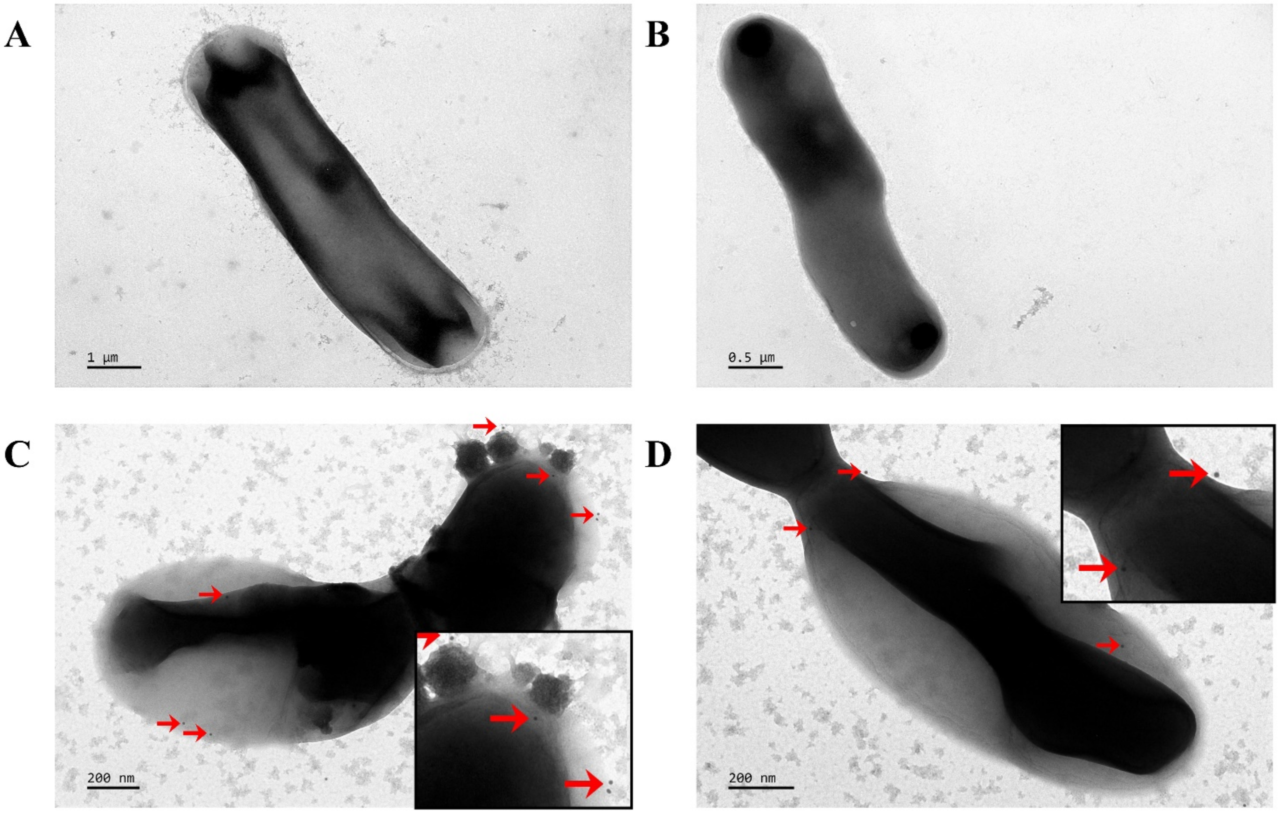

Supplementary Figure S3. The visualization of cell surface-localized FimM in *B. longum* BBMN68 by immunogold electron microscopy. (**A**, **B**) Control *B. longum* BBMN68 marked first with pre-immune serum and then with 10-nm gold particle-labeled protein A. (**C**, **D**) *B. longum* BBMN68 labeled first with anti-FimM serum and then with 10-nm gold particle-labeled protein A. Red arrows indicate the gold particle labeling of FimM proteins. Scale bars, 1 µm (**A**), 0.5 µm (**B**), and 200 nm (**C**, **D**).

(A)

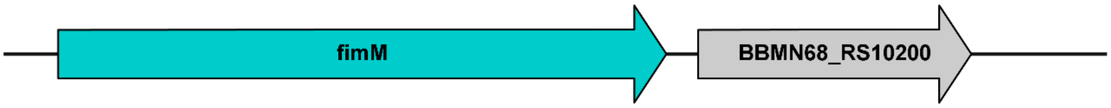

(B)

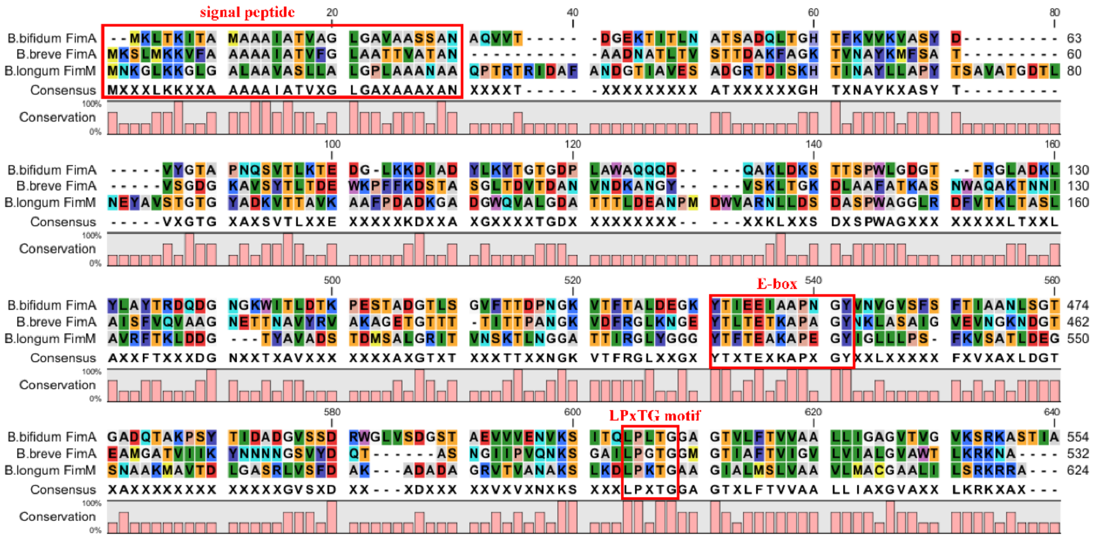

(C)

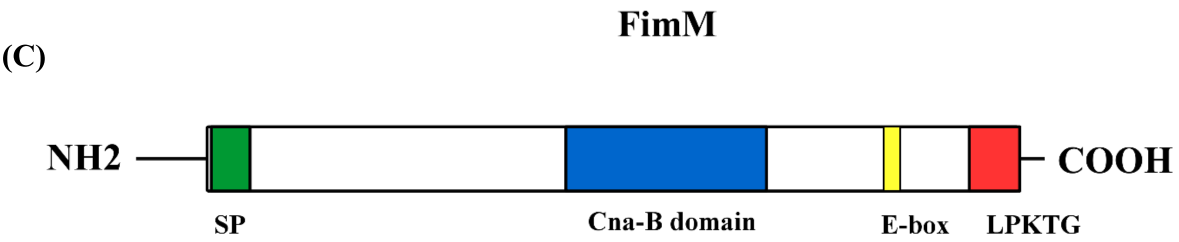

Supplementary Figure S4. (A) A Schematic representation of the genomic organization of *fimM* clusters identified from the BBMN68 genome sequence as published at NCBI; each arrow indicates an ORF, the size of which is proportional to the length of the arrow; aqua blue arrow indicates *fimM* gene, grey arrows indicate pseudogene. (B) Alignment of FimM in *B. longum BBMN68* and FimA in *B. bifidum* PRL2010 and *B. breve* UCC2003. (C) Schematic illustration of the domain organization for FimM protein is based on the deduced primary structure of the FimM ORF from BBMN68; FimM consist of one Cna-B domain, an E-box, with signal peptide (SP) and LPxTG motif sorting signals at the N- and C-terminals, respectively. The pictorial representation was done using Illustrator of Biological Sequences (IBS v1.0) (Liu et al., 2015). Alignments were obtained using ClustalX 2.1 (Larkin et al., 2007) with default settings and visualized in CLC Sequence Viewer 7.8.1.

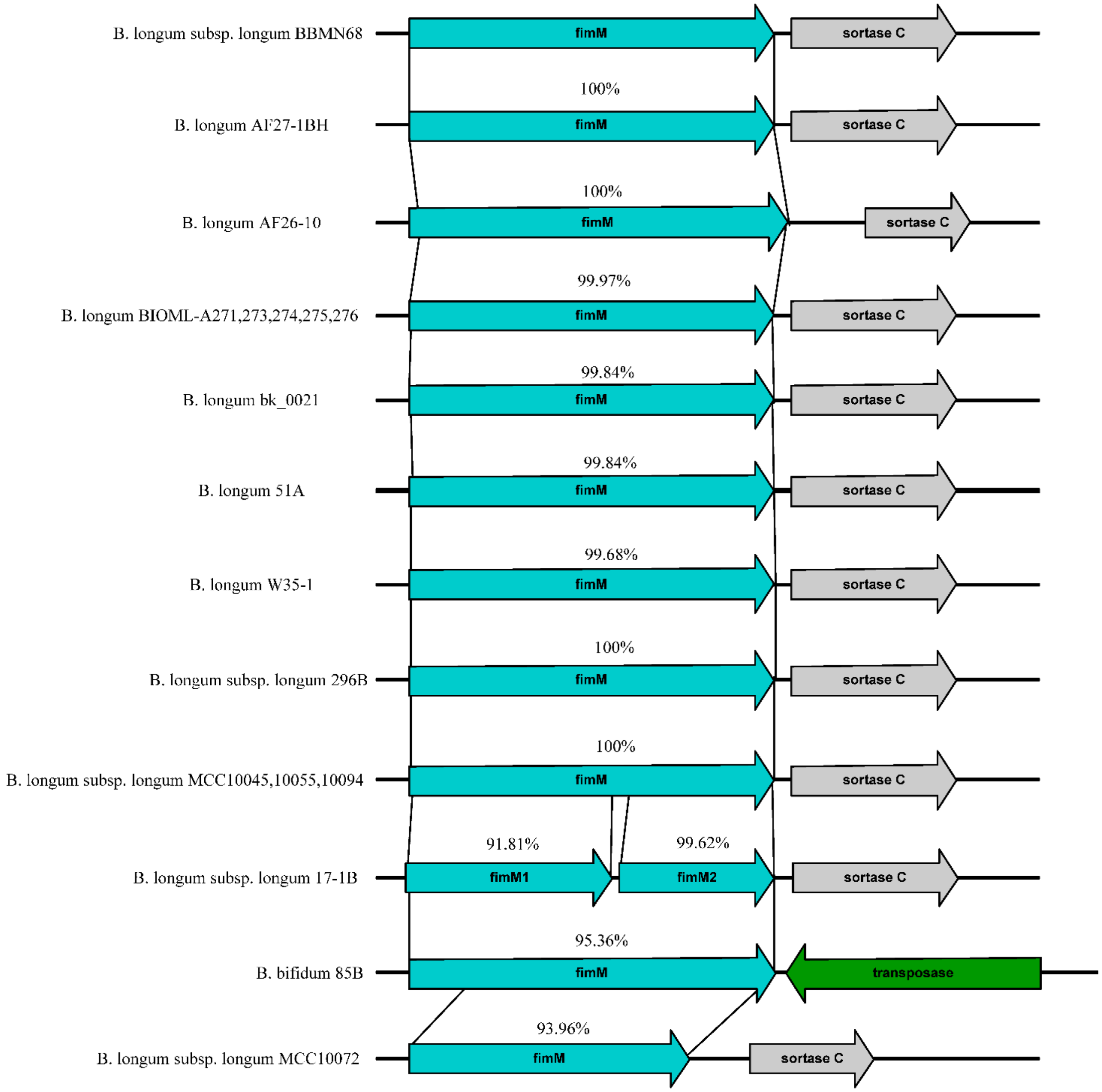

Supplementary Figure S5. Comparative *in silico* analysis of the genomic loci of *B. longum* AF27-1BH, AF26-10, BIOML-A271, 273,274, 275, 276, bk_0021, 51A, W35-1, *B. longum* subsp. *longum* BBMN68, 296B, MCC10045, 10055, 10094, 10072, 17-1B and *B. bifidum* 85B encoding *fimM* and adjacent genes by BLAST analysis; each arrow indicates an ORF, the size of which is proportional to the length of the arrow; aqua blue arrow indicates *fimM* gene, grey arrows indicate pseudogenes and green arrow indicates transposase gene; the amino acid identity of the relevant encoded proteins is indicated in percentages. The pictorial representation was done using Illustrator of Biological Sequences (IBS v1.0) (Liu et al., 2015).

1.2 Supplementary Tables

Supplementary Table S1 Bacterial strains and plasmids used in this study

Strain or plasmid	Relevant phenotype or genotype[a]	Source or reference
Bacterial strains		
B. longum BBMN68	Wild-type strain, isolated from feces from healthy centenarian	(Hao et al., 2011)
E. coli DH5α	F^-, φ80*lacZ* △M15, △ (*lacZYA-argF*) U169, *endA1*, *recA1*, *hsdR17*(rk^-, mk^+), *supE44*, λ^-, thi^{-1}, *gyrA96*, *relA1*, *phoA*	TianGen
E. coli BL21(DE3) LysS	F^-, *ompT*, *hsdSB* (rB^-, mB^-), *gal*, *dcm* (DE3), *pLysS*, Cm^r	TianGen
L. lactis NZ9000	*L. lactis* MG1363 *pepN:: nisRK*	(DeRuyter et al., 1996)
L. lactis NZCK	*L. lactis* NZ9000 harboring pNZ81481	This work
L. lactis NZ0860/NZlspA	*L. lactis* NZ9000 harboring pNZ00860	This work
L. lactis NZ2235/NZfimM	*L. lactis* NZ9000 harboring pNZ02235	This work
L. lactis NZ4435/NZslpA	*L. lactis* NZ9000 harboring pNZ04435	This work
L. lactis NZ4640/NZdppB3	*L. lactis* NZ9000 harboring pNZ04640	This work
L. lactis NZ5575/NZpotB	*L. lactis* NZ9000 harboring pNZ05575	This work
L. lactis NZ6090/NZaprE	*L. lactis* NZ9000 harboring pNZ06090	This work
L. lactis NZ6265/NZtadE	*L. lactis* NZ9000 harboring pNZ06265	This work
L. lactis NZ6270/NZtadF	*L. lactis* NZ9000 harboring pNZ06270	This work
L. lactis NZ7145/NZfimA	*L. lactis* NZ9000 harboring pNZ07145	This work
Plasmids		
pGEX-4T-1	Gene expression vector with Ptac; GST tag; Amp^r	GE Healthcare
pNZ8148	Gene expression vector with P_{nisA}; Cm^r	(DeRuyter et al., 1996)
pNZ81481	pNZ8148 derivative with modified MCS containing *Kpn*I, *Xba*I, *Sac*I, and *Hin*dIII sites	This work
pNZ00860/pNZlspA	pNZ81481 derivative containing *BBMN68_RS00860* gene	This work
pNZ02235/pNZfimM	pNZ81481 derivative containing *BBMN68_RS02235* gene	This work
pNZ04435/pNZslpA	pNZ81481 derivative containing *BBMN68_RS04435* gene	This work
pNZ04640/pNZdppB3	pNZ81481 derivative containing *BBMN68_RS04640* gene	This work
pNZ05575/pNZpotB	pNZ81481 derivative containing *BBMN68_RS05575* gene	This work
pNZ06090/pNZaprE	pNZ81481 derivative containing *BBMN68_RS06090* gene	This work
pNZ06265/pNZtadE	pNZ81481 derivative containing *BBMN68_RS06265* gene	This work
pNZ06270/pNZtadF	pNZ81481 derivative containing *BBMN68_RS06270* gene	This work
pNZ07145/pNZfimA	pNZ81481 derivative containing *BBMN68_RS07145* gene	This work

[a]Ptac, tac promoter; PnisA, nisA promoter; Cm^r, chloramphenicol resistance; Amp^r, ampicillin resistance; MCS, multiple cloning site.

Supplementary Table S2 Oligonucleotides and primers used in this study

Oligonucleotide or primer	Sequence (5' to 3') [a]
00860-F	CGGGGTACCATGACGAACCAACAGGGAC
00860-R	CCCAAGCTTGAAACGCTTACCCACCAAT
02235-F	CGGGGTACCATGAACAAAGGACTGAAG
02235-R	CCCAAGCTTCCAAGCGAGCGATGGAAAA
04435-F	CGGGGTACCATGAAGCACCTCTCCCAC
04435-R	CCCAAGCTTGCCTTTCAGGCCTTGTGG
04640-F	CGGGGTACCATGCGATTCGTGTTGAAAC
04640-R	CCCAAGCTTTCATGCGGTCACCTCCGT
05575-F	CGGGGTACCATGACCGCCGCAATCGCAC
05575-R	CCCAAGCTTTCATTGCCAACGTCCGGCC
06090-F	CGGGGTACCATGAAAAAGAAGAAGACTAT
06090-R	CTAGTCTAGAGATTGTAATGCTGGCTTTGG

06265-F	CGGGGTACCATGCGTTATGAGGAGGGA
06265-R	CCCAAGCTTGCTTTACGGTCCATTATTGTGA
06270-F	CGGGGTACCATGAAGACCATGACATGC
06270-R	CCCAAGCTTCAGCCAGCCGACCAGTTA
07145-F	CGGGGTACCATGAAGTCACTAATCAGG
07145-R	CCCAAGCTTCTCCTTCATGGGCCTTCC
fimM-GST-F	CCGGAATTCATGCAACCGACGCGGACACGC
fimM-GST-R	CCGCTCGAGTTAGGCGACGGTGACGCGGC
05880-F	GGACTAGTATGAGTTTCCATGTATCC
05880-R	TGCTCTAGACGAGCGAGTGGGACTAGCAG
05885-F	GGACTAGTATGCATCAATCAACACG
05885-R	TGCTCTAGATGACTCCGTTGCGGGTCAGG
07375-F	GGACTAGTATGACAACCAAACCATCG
07375-R	TGCTCTAGAGCCCTACAACTCGGAAATACA
07370-F	GGACTAGTATGGGAAAGCTGATACGA
07370-R	CCCAAGCTTAGTGGTGAAGTTCGGTAATGG
07380-F	GGACTAGTTTGGCGGCCTGCCTGACG
07380-R	TGCTCTAGATGTCTCCTCTGATTGTGGGTTT
07385-F	GGACTAGTATGGGTAAGTGGAAGAAAG
07385-R	TGCTCTAGACCTATTCGCACCACGATTTA
09380-F	CGGGGTACCGTGGCGATCGTTGCCGCC
09380-R	CCCAAGCTTCTCCGCAATCCGCAAATC
06495-F	GGACTAGTATGACCATCGAAAGCACT
06495-R	TGCTCTAGACCTCCAACGGCTCTTTACG
09410-F	CGGGGTACCATGACATCCCGTCAGGGC
09410-R	CCCAAGCTTTCACCTGCGGCGACGGGC
07430-F	CGGGGTACCATGAAGATAAACAATAAG
07430-R	CCCAAGCTTCGCAATCCACGCTACTCA
07365-F	GGACTAGTGTGAAGCATTGGAAGAAG
07365-R	TGCTCTAGAGGTTGCGCCTTCAGTTATG

[a]Restriction enzyme cutting sites are underlined (*Kpn*I, GGTACC; *Hin*dIII, AAGCTT; *Xba*I, TCTAGA; *EcoR*I, GAATTC; *Xho*I, CTCGAG; *Spe*I, ACTAGT).

Supplementary Table S3 Quantitative summary of proteins' subcellular localization predicted by PSORTdb

SCL[a]	NO. of proteins predicted by PSORTdb
Cellwall	25
Cytoplasmic	955
Cytoplasmic Membrane	507
Extracellular	16
Unknown	291
Unknown/multiple location	12
Total	1806

[a]SCL, subcellular location; Multiple location means more than one subcellular localization was predicted.

Supplementary Table S4 *Bifidobacterium* strains containing FimM homologs

**Bifidobacterial strains**	**Protein ID**
B. longum BBMN68	WP_013410854.1
B. longum AF27-1BH	RGQ71662.1
B. longum AF26-10	RGR17073.1
Unnamed *B. longum* strain	WP_013410854.1
Unnamed *B. longum* strain	WP_153188164.1
B. longum BIOML-A274	KAB6923263.1
B. longum BIOML-A275	KAB6923938.1
B. longum BIOML-A273	KAB6928322.1
B. longum BIOML-A271	KAB6928897.1
B. longum BIOML-A276	KAB6932694.1
B. longum bk_0021	RYT54833.1
Unnamed *B. longum* strain	WP_169051291.1
B. longum 51A	QCH30524.1
Unnamed *B. longum* strain	WP_147294974.1
B. longum W35-1	RDX16528.1
Unnamed *B. longum* strain	WP_165542379.1
Unnamed *B. longum* strain	WP_032746455.1
Unnamed *B. longum* strain	WP_032746456.1
B. longum subsp. *longum* 296B	OJS83597.1
B. longum subsp. *longum* MCC10045	TCE44971.1
B. longum subsp. *longum* MCC10055	TCE62973.1
B. longum subsp. *longum* MCC10094	TCF27775.1
B. longum subsp. *longum* MCC10072	TCE90246.1
B. longum subsp. *longum*17-1B	KEY21251.1
B. longum subsp. *longum*17-1B	KEY21252.1
B. bifidum 85B	KLN81709.1
B. gallinarum CACC 514	QAY32942.1

References:

DeRuyter, P., Kuipers, O.P., and DeVos, W.M. (1996). Controlled gene expression systems for *Lactococcus lactis* with the food-grade inducer nisin. *Appl. Environ. Microb.* 62, 3662-3667. doi: 10.1128/AEM.62.10.3662-3667.1996.

Hao, Y., Huang, D., Guo, H., Xiao, M., An, H., Zhao, L., Zuo, F., Zhang, B., Hu, S., Song, S., Chen, S., and Ren, F. (2011). Complete Genome Sequence of *Bifidobacterium longum* subsp. *longum* BBMN68, a New Strain from a Healthy Chinese Centenarian. *J. Bacteriol.* 193, 787-788. doi: 10.1128/JB.01213-10.

Larkin, M.A., Blackshields, G., Brown, N.P., Chenna, R., McGettigan, P.A., McWilliam, H., Valentin, F., Wallace, I.M., Wilm, A., Lopez, R., Thompson, J.D., Gibson, T.J., and Higgins, D.G. (2007). Clustal W and clustal X version 2.0. *Bioinformatics* 23, 2947-2948. doi: 10.1093/bioinformatics/btm404.

Liu, W., Xie, Y., Ma, J., Luo, X., Nie, P., Zuo, Z., Lahrmann, U., Zhao, Q., Zheng, Y., Zhao, Y., Xue, Y., and Ren, J. (2015). IBS: an illustrator for the presentation and visualization of biological sequences. *Bioinformatics* 31, 3359-3361. doi: 10.1093/bioinformatics/btv362.
